# Handgrip Strength Correlated with Falling Risk in Patients with Degenerative Cervical Myelopathy

**DOI:** 10.3390/jcm10091980

**Published:** 2021-05-05

**Authors:** Kathryn Anne Jimenez, Ji-Won Kwon, Jayeong Yoon, Hwan-Mo Lee, Seong-Hwan Moon, Kyung-Soo Suk, Hak-Sun Kim, Byung Ho Lee

**Affiliations:** Orthopedic Department, College of Medicine, Yonsei University, Seoul 03722, Korea; kathrynjimenez@yahoo.com (K.A.J.); KWONJJANNG@yuhs.ac (J.-W.K.); COFFEESOUND2@yuhs.ac (J.Y.); hwanlee@yuhs.ac (H.-M.L.); shmoon@yuhs.ac (S.-H.M.); sks111@yuhs.ac (K.-S.S.); haksunkim@yuhs.ac (H.-S.K.)

**Keywords:** cervical myelopathy, hand grip strength, falls, postoperative

## Abstract

Background: Few studies have investigated associations between hand grip strength (HGS) and the surgical outcomes of degenerative cervical myelopathy (DCM). Methods: This study was designed as a prospective observational study of 203 patients who had undergone fusion surgery for DCM. We divided the patients according to sex and HGS differences. Clinical outcome parameters, including HGS, a fall diary and four functional mobility tests (alternative step test, six-meter walk test, timed up and go test, and sit-to-stand test) were measured preoperatively, at 3 months and 1 year after surgery. Results: Mean patient ages were 59.93 years in the male group and 67.33 years in the female group (*p* = 0.000; independent *t*-test). The mean HGS of both hands improved significantly at postoperative 3 months and 1 year in all patients (*p* = 0.000 for both; ANOVA). In male patients, preoperative risk of falls was negatively correlated with HGS (*p* = 0.000). In female patients, pre- and postoperative risk of falls were correlated negatively with HGS (*p* = 0.000). The postoperative incidence of falls decreased in both groups (*p* = 0.000) Conclusions: Postoperative HGS in patients with DCM is correlated with postoperative falls and functional outcome differently, when comparing male and female patients, for predicting favorable outcomes and neurologic deficit recovery after surgery in DCM patients.

## 1. Introduction

Patients with spinal stenosis, either cervical and/or lumbar, are at an increased risk of falling [1,2]. Surgical treatment for both cervical and lumbar stenosis have been shown to decrease the risk of falling by improving physical performance, including walking and balancing [1,2,3,4,5]. There are many reported studies on prevalence, results of conservative or surgical treatment, gait patterns, hand dexterity–functional impairment and predictors of degenerative cervical myelopathy (DCM) [6,7,8,9,10,11,12,13,14,15,16,17,18]. A recent study reported that hand grip strength (HGS) might be a useful surrogate marker with which to predict the risk of falls and clinical outcomes in patients with lumbar stenosis [19]. 

Compared to lumbar stenosis, patients with DCM could have higher correlation between increased risk of falling and weakened HGS [20,21,22,23,24]. We also suspected that any observed correlations would differ according to sex. Our objectives in this investigation were to assess correlations for HGS with postoperative changes in the risk of falling and QoL in patients with DCM, separately for both men and women.

## 2. Materials and Methods

### 2.1. Subjects

This study was approved by the Institutional Review Board of the authors’ hospital (IRB No. 4-2020-1162). From March 2017 to August 2019, 203 patients who had undergone cervical spine surgery, including decompression and fusion procedure(s), for the treatment of DCM were included prospectively. All included patients had completed postoperative follow up for 1 year. All of the patients exhibited myelopathic symptoms, including clumsiness of the hand, poor hand coordination (e.g., difficulty with handwriting and using chopsticks), and walking difficulty, and had been recommended for surgical intervention by the management guidelines of DCM [25].

The exclusion criteria were as follows: comorbidity impairing physical function (e.g., history of cerebral infarction, cerebral palsy, Parkinson’s disease, spine surgery, head trauma, current/old cerebrovascular events (cerebral hemorrhage and cerebral infarct), and other neurodegenerative conditions or severe rheumatoid arthritis); bedridden status or full dependence on a wheelchair before surgery because of severe cervical myelopathy; and difficulty completing the questionnaire because of cognitive impairment. Furthermore, patients with severe osteo-arthropathic conditions that could cause knee and hip joint contracture affecting whole spinal sagittal balance were also excluded from the patient pool [26]. No patients were diagnosed with hand- or wrist-related diseases, such as carpal tunnel syndrome and tardy ulnar nerve palsy.

The major included diagnoses were cervical stenosis with myelopathy (DCM) (135 patients), ossified posterior longitudinal ligament (44 patients), and herniated cervical disc with myelopathy (24 patients).

Patients were treated with decompression and instrumented fusion (anterior plate-screw system; ZEVO™ plate and screw system; Medtronic Sofamor Danek, Memphis, TN, USA) for anterior surgery or a posterior screw-rod system (Poseidon, Medyssey, Jecheon, Korea) for combined anterior-posterior surgery. Cervical allograft allospacers (Cornerstone^TM^; ASR Medtronic Sofamor Danek, Memphis, TN, USA) were utilized for anterior fusion surgery. For posterior surgery, local autologous and demineralized bone matrix grafts (Bongener™; CG-BIO, Seoul, Korea) were used. The surgically treated level and other demographic data, including the presence of spinal cord signal changes on MRI scans, are presented in Table 1. 

### 2.2. Outcome Measures

For all enrolled patients, the Neck Disability Index (NDI, higher scores reflecting worse functional status), Euro-QoL Visual Analog Scale (VAS, higher scores indicating better QoL), modified Japanese Orthopedic Association (JOA) score (higher scores representing better functional status), modified JOA grade (16~17 = Grade 0; 12~15 = Grade 1; 8~11 = Grade 2; 0~7 = Grade 3, with higher grades reflecting worse functional status), modified frailty index (mFi) (higher index scores indicating greater frailty), and HGS of both hands were measured and recorded preoperatively and at 3 months and 1 year after surgery [27,28,29,30,31].

### 2.3. HGS Measurement

HGS was measured using a Jamar Plus+ hand grip dynamometer (Global Medical Devices, Maharashtra, India). Patients were instructed to squeeze the handle as hard as possible for 3 s, and the maximum contractile force (lbs.) was recorded. The tests were performed three times on both hands. The highest value of the three repeated measurements was used for analysis [30]. The HGS of patients was measured preoperatively and at 3 months and 1 year after surgery. Considering basic physical differences, the patient groups were divided into male and female groups and compared.

### 2.4. Assessment of the Risk of Falling Using Four Functional Mobility Tests and an Actual Fall Diary

To evaluate the risk of falling, four functional mobility tests were used: the alternate-step test (AST), the six-meter-walk test (SMT), the sit-to-stand test (STS), and the timed up and go test (TUGT). These four tests have been validated in previous studies [2]. Additionally, a fall diary was given to patients or caregivers who were encouraged to record every fall and fall-related neurologic deficit and to report it to the clinical research coordinator when they visited the outpatient clinic for regular follow up at 3 months and 1 year postoperatively [4].

### 2.5. Statistical Analysis

Basic statistical tests, independent t-test, analysis of variance (ANOVA), and chi-squared test were used to evaluate whether the differences between the male and female surgery groups in terms of QoL, the four functional mobility tests, and other demographic data were statistically significant. Multiple linear regression analyses among measured HGS, falls, signal changes of the spinal cord, NDI, EQ-VAS, fall-related functional mobility tests, and other values were performed. All statistical analyses were performed using the SPSS 22.0 statistics package (SPSS, Inc., Chicago, IL, USA). *p* values < 0.05 were considered statistically significant.

## 3. Results

Mean patient ages were 59.93 years in the male group (range, 52–85 years) and 67.33 years in the female group (range, 52–86 years) (*p* = 0.000; independent *t*-test). Other demographic comparisons, including sex and body mass index (BMI), are shown in Table 1. All parameters differed significantly between the male and female groups.

### 3.1. Functional Mobility Test Results and Actual Falls

**Figure 1:**
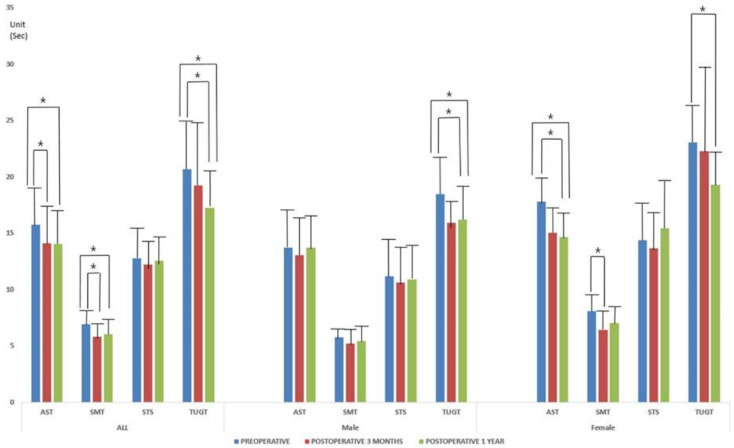
The pre- and postoperative values of the four functional tests depending on the patient groups. Preoperative measures were improved significantly at postoperative 3 months and 1 year in the male and female patient groups (*p* = 0.000 for all; ANOVA). * indicates the statistical difference between measures.

The pre- and postoperative values of the four functional tests in the male and female groups are presented in Table 2. In both groups, preoperative measures improved significantly at postoperative 3 months and 1 year, except STS (*p* = 0.000, 0.000, and 0.000 for AST, SMT, and TUGT, respectively; ANOVA; Figure 1). All measures were significantly different between the male and female groups, except preoperative falls and AST, at postoperative 1 year.

The average number of actual falls per patient among all patients during follow up was 0.48 ± 1.82 in the preoperative group, 0.09 ± 0.37 at postoperative 3 months, and 0.09 ± 0.43 at postoperative 1 year (*p* = 0.000; ANOVA). A significant difference was found in the distribution of non-fallers and fallers (single-time and multiple fallers) between preoperative and postoperative follow up among all patients (*p* = 0.005; chi-squared test). During follow up, no neurology deterioration related to falls was recorded.

### 3.2. QoL Outcomes: EQ-VAS, NDI, and mJOA Score and Grade

Mean EQ-VAS scores were 48.62 ± 23.79 preoperatively, 58.55 ± 22.48 at 3 months postoperatively, and 60.31 ± 17.88 at 1 year postoperatively in all patients (*p* = 0.000; ANOVA). Mean NDI values in all patients were 17.31 ± 7.77 preoperatively, 14.82 ± 5.73 at 3 months postoperatively, and 12.68 ± 9.06 at 1 year postoperatively (*p* = 0.000; ANOVA). Other mJOA scores and grade measures also improved after surgery in all patients (*p* = 0.013 and 0.010, respectively; ANOVA). The results are presented in Table 3.

### 3.3. HGS

**Figure 2:**
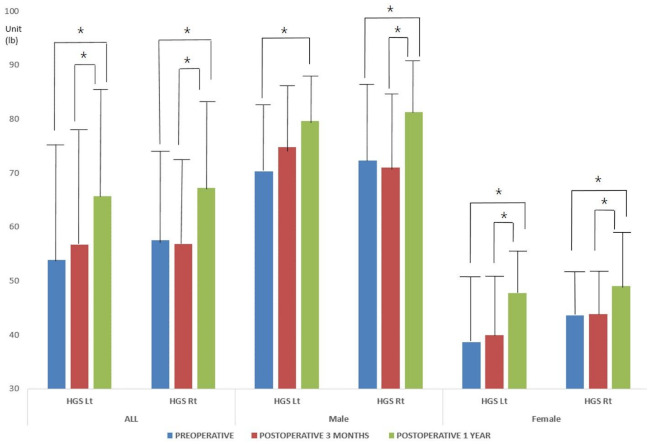
The mean HGS of both hands improved significantly at postoperative 3 months and 1 year compared with the preoperative measures in all patient groups (*p* = 0.000 for all; ANOVA). * indicates the statistical difference between measures.

The mean HGS of both hands improved significantly at postoperative 3 months and 1 year, compared with the preoperative measures, in all patients (*p* = 0.000 for both; ANOVA) (Figure 2 and Table 4). A significant difference was found between the male and female groups for every measure (*p* = 0.000; independent *t*-test).

### 3.4. Multiple Regression Analyses of Parameters Associated with Falls and Fall-Related Mobility Tests

In male patients, preoperative falls were correlated positively with symptom duration (beta ± standard error = 0.003 ± 0.001, *p* = 0.000) and mFi (beta ± standard error = 0.362 ± 0.043, *p* = 0.000) and negatively with EQ-VAS (−0.002 ± 0.001) and HGS (beta ± standard error = −0.004 ± 0.001, *p* = 0.000). Falls at postoperative 3 months and 1 year were not correlated with any parameter.

In female patients, preoperative falls were correlated negatively with mJOA score (beta ± standard error = −0.057 ± 0.015, *p* = 0.000) and HGS (beta ± standard error = −0.035 ± 0.005, *p* = 0.000). At postoperative 3 months, number of falls was positively correlated with mFi (beta ± standard error = 0.246 ± 0.017, *p* = 0.000) and NDI (beta ± standard error = 0.050 ± 0.006, *p* = 0.000). Fall measures at 12 months were positively correlated with NDI (beta ± standard error = 0.066 ± 0.000, *p* = 0.000) and WC (beta ± standard error = 0.046 ± 0.000, *p* = 0.000) and negatively with HGS (beta ± standard error = −0.049 ± 0.000, *p* = 0.000). Other correlations with functional mobility tests are listed in Table 5: Correlations between parameters not presented in Table 5 lacked statistical significance. Additionally, univariate linear regression analyses were presented in the Table S1.

## 4. Discussion

Surgical treatment for DCM is associated with improvements in functional, disability-related, and QoL outcomes and reduced incidences of both falls and fall-related deterioration of subjective symptoms [5,32,33]. Compared with lumbar stenosis, the lack of data on DCM patients and the related risk of falls therein makes it difficult to predict surgical outcomes and postoperative rates of improvement in preoperative neurologic deficits. Additionally, prior studies that have characterized grip strength in association with myelopathic symptoms have presented mixed evidence with postoperative improvement, or no difference [34,35,36].

Compared with a recently published lumbar stenosis study, the present study confirmed differences in correlations between male and female sex and the postoperative risk of falling [19]. The previous study excluded cervical stenosis patients with upper-extremity motor deficits to focus on the sarcopenic conditions of the patients [37]. The present study focused on cervical myelopathy-related HGS weakness and postoperative functional changes according to sex. As expected, differences between the male and female groups were observable. Meanwhile, different from other available studies, all of the enrolled patients developed cervical myelopathic symptoms, and more than half also showed spinal cord signal changes (65.5%; 133/203). We confirmed that the spinal cord signal changes were not necessarily correlated with actual falls and other outcomes, such as functional mobility tests and QoL (Table 5), and the direction of correlations varied from positive to negative depending on the measured time and the sex, a finding that is consistent with the literature [38]. Healing of the spinal cord after surgical decompression is based on the intrinsic ability of the spinal cord to heal itself. Thus, the pre-operative health of the cord is paramount to post-operative improvement [39]. For the enrolled male and female patients in the present study, preoperative status, including the general condition and duration of symptoms (Table 1), could differ, and these could affect the observed variations in correlations with fall and fall-related parameters. Although there was a negative correlation between postoperative fall-related functional tests and HGS in female patients, it was smaller than that in the male patients in this study.

Along with HGS, the present study demonstrated sex differences in the recovery of QoL reflected in the outcomes and related functional mobility results. For male patients, because baseline HGS and muscle strength are much greater than those in female patients, a higher increase in HGS was expected postoperatively. Although a lesser amount of recovery of HGS and related function was observed in the female group by postoperative 3 months, the larger delayed recovery between postoperative 3 months and 1 year (Table 4) could lead the patients and medical team to encourage functional rehabilitation to improve muscle strength and lower the risk of falling up to postoperative 1 year. [40].

In a study by Kalsi-Ryan et al., [14,15] a more specific hand assessment study was suggested. Unfortunately, in this study, the patients were enrolled from March 2017 to August 2019, and therefore the specific test was not yet available. The authors believe that the hand assessment study would be better to describe upper extremity function in DCM patients in future studies.

The surgical effect of decompression in patients with DCM could differ in relation to a variety of factors. Since HGS improved after surgical decompression, the recovery of HGS was not only related to preoperative HGS but also to the overall functional outcome originating from compressive myelopathy-related pyramidal tract dysfunction [36]. Improved concordant motor function and muscle coordination with the resolution of myelopathy symptoms postoperatively elicited better functional mobility tests related to the risk of falling and actual falls [5,32].

The key findings of the present study are the following: postoperative HGS may be correlated with postoperative falling and functional outcomes differently in male and female patients. Meanwhile, surgical intervention for DCM significantly reduced the incidence rate of falls to less than 40% of the preoperative rate. The incidence of falls decreased significantly from 17.2% (35/203) to 6.8% (14/203) after surgery. Frequent falling is one of the most common symptoms in patients with DCM, and our analysis revealed that the incidence of both actual falls and multiple falls decreased significantly during postoperative follow up (Table 2). The decrease in actual falls during follow-up, however, made multiple regression analyses thereof in relation to other parameters impossible.

In another study, the incidence of postoperative falls peaked at 5 to 6 months after surgery, likely because many patients may have increased their daily walking activity during this period, leading to a transiently increased fall rate [5]. However, only a limited number of patients fell during follow up and no aggravation of symptoms and related fractures were reported in the present study. This finding could be explained by the peri- and postoperative fall prevention education program provided by our institution to emphasize the risk and caution of postoperative falls to patients and caregivers during admission and at every outpatient clinic follow up, based on previous publications [2,3,4,19].

Another possible reason for the decreased number of falls during follow-up could be the low BMI (mean: 24.30 ± 3.82 kg/m^2^) of the enrolled patients. A higher BMI is an independent risk factor for falls, and an association between increasing BMI (ranging from 25.0 to 29.9 kg/m^2^ and 30.0 kg/m^2^ and higher) and the risk of falls has been reported [41]. However, no significant association was found between increasing BMI and fall-related injury in the present study: correlations between functional mobility tests and BMI are presented in Table 5.

Our study had several strong points compared with previous studies. We evaluated a comprehensive range of risk factors, including the duration of symptoms and comorbidity. As the general condition of the patients is related to the preoperative and postoperative recovery of function, the overall condition of the patients is an important factor [42,43]. Additionally, we included more severe spondylotic myeloradiculopathic cases that had undergone combined anterior–posterior surgery [44,45,46], and as such the rate of combined anterior–posterior surgeries was much higher than that in another study [5]. Moreover, we report not only actual falls but also the objective measures of functional mobility tests and HGS, which all affect patient subjective symptoms.

A limitation of the present study was that the radiologic factors for the risk of falling were not reported at the same time. However, regarding the functional evaluation in the present study, all parameters, including mFi and HGS, would help clarify the postoperative recovery patterns of DCM patients. The results concerning radiologic evaluation and analyses are now being prepared for a future study. Despite these limitations, this is the first study to analyze correlations between HGS and the risk of falls in relation to functional tests and actual falls, as well as QoL, in DCM.

## 5. Conclusions

Postoperative HGS in patients with DCM is correlated with postoperative falls and functional outcome differently in male and female patients. Altogether, our results suggest that postoperative HGS could be used as a surrogate marker for predicting favorable outcomes and neurologic deficit recovery after surgery in DCM patients, provided careful consideration in given to sexual differences therein.

## Figures and Tables

**Table 1:** Demographic parameters of the enrolled patients.

	All	Male	Female	*p* Value
	(*N* = 203)	(*N* = 98)	(*N* = 105)	
Age (years)	63.76 ± 10.56	59.93 ± 10.26	67.33 ± 9.56	0.000
Symptom duration (months)	37.17 ± 40.87	29.92 ± 33.22	46.73 ± 44.98	0.000
Body mass index (kg/m^2^)	24.30 ± 3.82	23.69 ± 2.45	24.86 ± 4.71	0.026
Waist circumference (cm)	89.43 ± 10.0	88.82 ± 9.30	90.00 ± 1.04	N.S
Modified frailty index	1.37 ± 1.27	1.14 ± 1.13	1.60 ± 1.36	0.010
Smoker:non-smoker *	42:161	35:63	7:98	0.000
Spinal cord signal change (+):(−) *	133:70	77:21	56:49	0.000
Operation length (fusion level) *	2.96 ± 0.93	2.85 ± 0.91	3.06 ± 0.93	0.000
1 level	7 (3.4%)	0	7 (6.7%)	
2 level	63 (31%)	42 (42.9%)	21 (20%)	
3 level	70 (34.5%)	35 (35.7%)	35 (33.3%)	
4 level	56 (27.6%)	14 (14.3%)	42 (40.0%)	
5 level	7 (3.4%)	7 (7.1%)	0	
Surgery type *				0.002
Anterior	91 (44.8%)	56 (57.1%)	35 (33.3%)	
Posterior	42 (20.7%)	14 (14.3%)	28 (26.7%)	
Combined anterior-posterior	70 (34.5%)	28 (28.6%)	42 (40.0%)	

Statistical analyses were performed by independent *t*-test and * chi-squared test.

**Table 2:** Comparison of functional test results between male and female patients.

	All	Males	Females	*p* Value
Preoperative (unit: seconds)				
Alternate-Step Test	15.74 ± 4.38	13.71 ± 3.2	17.78 ± 4.46	0.000
Six-Meter-Walk Test	6.91 ± 2.82	5.74 ± 2.52	8.08 ± 2.61	0.000
Sit-to-Stand test	12.78 ± 3.82	11.18 ± 3.46	14.38 ± 3.49	0.000
Time Up and Go Test	20.66 ± 5.12	18.47 ± 5.18	23.02 ± 3.87	0.000
Actual fall *				
No fall:fall	168:35	84:14	84:21	NS
Single:multiple	20:15	8:6	12:9	NS
Postoperative 3 months(unit: seconds)				
Alternate-Step Test	14.12 ± 3.52	13.06 ± 3.72	15.04 ± 3.07	0.000
Six-Meter-Walk Test	5.84 ± 1.96	5.19 ± 0.85	6.40 ± 2.42	0.000
Sit-to-Stand test	12.24 ± 3.55	10.63 ± 2.10	13.64 ± 3.94	0.000
Time Up and Go Test	19.22 ± 7.70	15.92 ± 2.97	22.29 ± 9.32	0.000
Actual fall *				
No fall:fall	189:14	98:0	91:14	0.000
Single:multiple	10:4	0	10:4	0.000
Postoperative 1 year (unit: seconds)				
Alternate-Step Test	14.05 ± 3.35	13.69 ± 3.08	14.65 ± 3.71	NS
Six-Meter-Walk Test	6.02 ± 1.98	5.42 ± 1.23	7.02 ± 2.53	0.000
Sit-to-Stand test	12.57 ± 5.03	10.87 ± 1.95	15.42 ± 7.00	0.000
Time Up and Go Test	17.23 ± 4.77	16.21 ± 5.06	19.27 ± 3.33	0.000
Actual fall *				
No fall:fall	189:14	96:2	93:12	0.000
Single:multiple	12:2	1:1	11:1	0.000

Statistical analyses were performed by independent *t*-test and * chi-squared test. NS, not significant.

**Table 3:** Comparison of functional test results between male and female patients.

	All	Males	Females	*p* Value
Preoperative				
Modified JOA score	9.51 ± 3.04	10.92 ± 3.00	8.20 ± 2.41	0.000
Modified JOA grade *				
Grade 0:1:2:3	7:49:98:49	7:35:42:14	0:14:58:35	0.000
Neck Disability Index	17.31 ± 7.77	14.85 ± 6.67	19.60 ± 8.05	0.000
Euro-QoL Visual Analog Scale	48.62 ± 23.79	50.71 ± 18.40	46.66 ± 27.86	NS
Postoperative 3 months				
Modified JOA score	11.58 ± 3.14	12.35 ± 3.1	10.86 ± 3.02	0.001
Modified JOA grade *				
Grade 0:1:2:3	28:77:63:35	21:35:35:7	7:42:28:28	0.001
Neck Disability Index	14.82 ± 5.73	14.21 ± 7.13	15.40 ± 3.96	NS
Euro-QoL Visual Analog Scale	58.55 ± 22.48	62.71 ± 25.68	54.66 ± 18.29	0.011
Postoperative 1 year				
Modified JOA score	12.61 ± 3.50	12.444 ± 2.38	12.71 ± 3.61	NS
Modified JOA grade *				
Grade 0:1:2:3	30:79:64:30	22:34:35:7	8:45:29:23	0.001
Neck Disability Index	12.68 ± 9.06	12.66 ± 10.80	12.71 ± 6.26	NS
Euro-QoL Visual Analog Scale	60.31 ± 17.88	62.22 ± 16.13	57.85 ± 19.81	NS

Statistical analyses were performed by independent *t*-test and * chi-squared test between the male and female groups. NS, not significant.

**Table 4:** Hand grip strength measurements.

(Unit: lbs.)	All	Males	Females	*p* Value
Preoperative				
HGS Lt	53.90 ± 23.83	70.30 ± 19.46	38.59 ± 16.11	0.000
HGS Rt	57.51 ± 22.43	72.35 ± 21.96	43.66 ± 11.26	0.000
Postoperative 3 months				
HGS Lt	56.78 ± 24.47	74.79 ± 21.39	39.97 ± 12.07	0.000
HGS Rt	56.97 ± 21.42	71.01 ± 20.48	43.86 ± 11.88	0.000
Postoperative 1 year				
HGS Lt	65.72 ± 21.50	79.60 ± 16.93	47.86 ± 10.95	0.000
HGS Rt	67.23 ± 23.21	81.32 ± 18.75	49.12 ± 14.02	0.000

Statistical analyses were performed by independent *t*-test comparing the male and female groups.

**Table 5:** Multiple regression analyses of fall-related functional mobility tests.

Males								
Variables	AST		SMT		STS		TUGT	
	Beta ± S.E	*p* Value	Beta ± S.E	*p* Value	Beta ± S.E	*p* Value	Beta ± S.E	*p* Value
Preoperative								
NDI	0.330 ± 0.016	0.000	0.325 ± 0.001	0.000	0.460 ± 0.000	0.000	0.545 ± 0.014	0.000
AGE	0.168 ± 0.010	0.000	−0.084 ± 0.001	0.000	0.023 ± 0.000	0.000		
mFI	1.988 ± 0.145	0.000	1.605 ± 0.008	0.000	−0.386 ± 0.003	0.000	2.370 ± 0.143	0.000
BMI	0.681 ± 0.063	0.000	0.989 ± 0.003	0.000	1.299 ± 0.001	0.000	2.029 ± 0.048	0.000
Operation length	0.955 ± 0.095	0.000	1.153 ± 0.006	0.000	4.326 ± 0.002	0.000	3.998 ± 0.128	0.000
* Modified JOA grade			−0.711 ± 0.015	0.000	4.467 ± 0.006	0.000	3098 ± 0.278	0.000
Postoperative 3 months								
NDI	−0.387 ± 0.024	0.000	0.190 ± 0.016	0.000			−0.127 ± 0.007	0.000
AGE	0.326 ± 0.007	0.000			0.224 ± 0.002	0.000	0.227 ± 0.004	0.000
mFI			0.438 ± 0.173	0.013	−2.639 ± 0.024	0.000	1.164 ± 0.055	0.000
Smoking	1.199 ± 0.115	0.000	1.647 ± 0.068	0.000	4.264 ± 0.023	0.000	−4.956 ± 0.041	0.000
BMI	2.280 ± 0.051	0.000			0.053 ± 0.005	0.000	2.003 ± 0.021	0.000
Operation length	3.169 ± 0.152	0.000			0.672 ± 0.008	0.000	1.452 ± 0.036	0.000
* Modified JOA grade	6.866 ± 0.256	0.000	1.309 ± 0.209	0.000	9.980 ± 0.045	0.000		
Postoperative 1 year								
mFI	3.040 ± 0.004	0.000			2.156 ± 0.022	0.000		
Smoking	0.275 ± 0.009	0.000	−0.079 ± 0.000	0.000	1.331 ± 0.031	0.000		
HGS	−0.051 ± 0.000	0.000			−0.129 ± 0.001	0.000	−0.024 ± 0.001	0.000
Symptom duration	0.063 ± 0.000	0.000	0.006 ± 0.000	0.000	0.042 ± 0.000	0.000	0.074 ± 0.00	0.000
* Modified JOA grade			1.836 ± 0.000	0.000		.	7.679 ± 0.055	0.000
**Females**								
**Variables**	**AST**		**SMT**		**STS**		**TUGT**	
	**Beta ± S.E**	***p* Value**	**Beta ± S.E**	***p* Value**	**Beta ± S.E**	***p* Value**	**Beta ± S.E**	***p* Value**
Preoperative								
NDI	0.539 ± 0.018	0.000	0.791 ± 0.010	0.000	0.890 ± 0.004	0.000	−0.275 ± 0.026	0.000
AGE	0.812 ± 0.034	0.000	1.162 ± 0.018	0.000	0.899 ± 0.005	0.000	−0.620 ± 0.044	0.000
WC	0.271 ± 0.018	0.000	0.387 ± 0.010	0.000		0.000		
Cord signal change	2.674 ± 0.251	0.000	−2.885 ± 0.136	0.000	5.966 ± 0.042		6.574 ± 0.184	0.000
* Modified JOA score	−2.500 ± 0.051	0.000	−2.578 ± 0.028	0.000	−1.309 ± 0.011	0.000	0.498 ± 0.086	0.000
HGS			−0.725 ± 0.014	0.000	−0.035 ± 0.003	0.000	−0.300 ± 0.018	0.000
Operation length	4.793 ± 0.075	0.000	2.934 ± 0.041	0.000	3.301 ± 0.013	0.000	−0.825 ± 0.149	0.000
Postoperative 3 months								
NDI	−1.414 ± 0.080	0.000	−0.079 ± 0.034	0.022	0.412 ± 0.038	0.000	0.831 ± 0.278	0.004
AGE	0.036 ± 0.013	0.006	0.132 ± 0.015	0.000	0.141 ± 0.006	0.000	0.654 ± 0.055	0.000
WC	0.425 ± 0.031	0.000	0.177 ± 0.028	0.000	0.612 ± 0.017	0.000	1.132 ± 0.100	0.000
* Modified JOA score	−1.528 ± 0.118		−0.725 ± 0.163	0.000	−2.569 ± 0.070	0.000	−2.606 ± 0.553	0.000
HGS	−0.335 ± 0.052	0.000			−0.168 ± 0.012	0.000		
BMI		0.000	−0.066 ± 0.063	0.000	−0.989 ± 0.029	0.000	−1.954 ± 0.227	0.000
Postoperative 1 year								
* Modified JOA score	−1.474 ± 0.055	0.000	−1.616 ± 0.000	0.000	−3.734 ± 0.050	0.000	0.837 ± 0.008	0.000
HGS	−0.269 ± 0.016	0.000	0.010 ± 0.000	0.000				
Operation length			−0.974 ± 0.001	0.000	−5.774 ± 0.170	0.000	−1.758 ± 0.017	0.000
* Modified JOA grade	−6.145 ± 0.232	0.000	−6.533 ± 0.002	0.000	−13.403 ± 0.277	0.000		

* modified JOA grade (16~17 = Grade 0, 12~15 = Grade 1, 8~11 = Grad 2, 0~7 = Grade 3, higher grades reflect worse functional status); Neck Disability Index (NDI) (=higher scores indicate worse functional status), Euro-QoL Visual Analog Scale (VAS) (=higher scores represent better QoL status), modified Japanese orthopedic association (JOA) score (=higher scores reflect better functional status).

## Data Availability

Not applicable.

## Supplementary Materials

The following are available online at https://www.mdpi.com/article/10.3390/jcm10091980/s1, Table S1: Pearson correlation analysis of HGS.
