# Supplementary material for: Handgrip Strength Correlated with Falling Risk in Patients with Degenerative Cervical Myelopathy
Source: J Clin Med. 2021 May 5;10(9):1980. doi: 10.3390/jcm10091980 (PMC8125546; doi:10.3390/jcm10091980)
Supplement: Supplementary file 1 [file jcm-10-01980-s001.zip › jcm-1183339-supplementary.pdf]

Table S1. Pearson correlation analysis of HGS.

		Preoperative											
		Cord signal	age	Fall	NDI	mJOA score	mJOA grade	Fried Frailty model	EQ-VAS	AST	SMT	STS	TUGT
All	Coefficient (*r*)	0.147	−0.419	−0.320	−0.383	0.400	−0.301	0.693	0.227	−0.502	−0.423	−0.448	−0.526
	p-value	0.036	0.000	0.000	0.000	0.000	0.001	0.000	0.001	0.000	0.000	0.000	0.000
Male	Coefficient (*r*)	NS	−0.304	−0.367	−0.205	0.372	−0.283	0.289	0.267	−0.328	−0.359	−0.423	−0.218
	p-value	NS	0.002	0.000	0.043	0.000	0.000	0.004	0.008	0.001	0.000	0.000	0.031
Female	Coefficient (*r*)	-0.538	−0.277	−0.367	−0.436	0.608	−0.318	0.798	0.549	−0.287	−0.364	−0.335	−0.674
	p-value	0.000	0.004	0.000	0.000	0.000	0.000	0.000	0.000	0.004	0.000	0.001	0.000
		Postoperative 3 months											
		Cord signal	Age	Fall	NDI	mJOA score	mJOA grade	Frailty-Fried model	EQ-VAS	AST	SMT	STS	TUGT
All	Coefficient (r)	NS	−0.474	−0.240	−0.149	0.390	−0.408	0.539	0.203	−0.350	−0.364	−0.419	−0.297
	p-value	NS	0.000	0.001	0.034	0.000	0.000	0.000	0.040	0.000	0.000	0.000	0.000
Male	Coefficient (r)	NS	−0.308	NS	−0.183	0.394	−0.435	NS	NS	NS	−0.629	−0.428	
	p-value	NS	0.002	NS	0.071	0.000	0.000	NS	NS	NS	0.000	0.000	
Female	Coefficient (r)	−0.553	−0.427	NS	−0.216	0.321	−0.306	0.429	NS	−0.505	NS	NS	NS
	p-value	0.000	0.000	NS	0.027	0.001	0.001	0.000	NS	0.000	NS	NS	NS
		Postoperative 1 year											
		Cord signal	age	Fall	NDI	mJOA score	mJOA grade	Frailty-Fried model	EQ-VAS	AST	SMT	STS	TUGT
ALL	Coefficient (r)	0.693	−0.417	−0.363	−0.182	0.577	−0.713	0.385	0.194	−0.553	−0.665	−0.617	−0.573
	p-value	0.000	0.000	0.000	0.045	0.000	0.000	0.000	0.041	0.000	0.000	0.000	0.000
Male	Coefficient (r)	0.443	NS	NS	−0.313	0.300	−0.569	NS	0.426	−0.879	−0.891	−0.885	−0.670
	p-value	0.000	NS	NS	0.013	0.017	0.000	NS	0.000	0.000	0.000	0.000	0.000
Female	Coefficient (r)	NS	−0.516	NS	NS	0.632	−0.508	NS	NS	−0.682	−0.679	−0.608	−0.327
	p-value	NS	0.000	NS	NS	0.000	0.000	NS	NS	0.000	0.000	0.000	0.050
